# Dancing to a different tune, can we switch from chemical to biological nitrogen fixation for sustainable food security?

**DOI:** 10.1371/journal.pbio.3001982

**Published:** 2023-03-14

**Authors:** Min-Yao Jhu, Giles E. D. Oldroyd

**Affiliations:** Crop Science Centre, Department of Plant Sciences, University of Cambridge, Cambridge, United Kingdom

## Abstract

Our current food production systems are unsustainable, driven in part through the application of chemically fixed nitrogen. We need alternatives to empower farmers to maximise their productivity sustainably. Therefore, we explore the potential for transferring the root nodule symbiosis from legumes to other crops. Studies over the last decades have shown that preexisting developmental and signal transduction processes were recruited during the evolution of legume nodulation. This allows us to utilise these preexisting processes to engineer nitrogen fixation in target crops. Here, we highlight our understanding of legume nodulation and future research directions that might help to overcome the barrier of achieving self-fertilising crops.

## Abstract 2

To achieve sustainable crop production, we need to reduce our dependency on chemical nitrogen fertilizers. This Unsolved Mystery looks at whether it is possible to engineer plants to biologically fix their own nitrogen and create self-fertilizing crops.

## Introduction

The availability of reactive sources of Nitrogen (N) is one of the major limiting factors for crop production [1]. Since the onset of the Green Revolution [2], this limitation has been overcome with the application of inorganic fertilisers, leading to massive improvements in agricultural output. This agricultural revolution impacted farmers in high- and middle-income countries, but for some small-holder farmers in low-income countries, these expensive interventions are out of reach, leading to sizable yield gaps [1,3,4]. Agriculture and deforestation are responsible for about 19% of all greenhouse gas emissions [5], and chemical nitrogen fertiliser usage accounts for a significant proportion of the greenhouse gas emissions derived from agriculture [6], because of the massive energy usage required for its production and denitrification that results from its application [7,8]. Additionally, chemical nitrogen fertiliser usage is the principal source of agricultural pollution, with detrimental impacts on biodiversity in nearby terrestrial ecosystems and distant aquatic ecosystems [6,9]. Addressing the dependence on inorganic fertilisers goes to the heart of tackling sustainability and equity in global food production.

Nitrogen itself is not limiting: Much of our atmosphere is molecular dinitrogen, an extremely inert form of this element that is inaccessible to most organisms. Diazotrophic bacteria are the only organisms on the planet able to undergo biological nitrogen fixation, the conversion of nonreactive dinitrogen to the reactive form ammonia. The enzyme that facilitates this conversion, nitrogenase, is competitively inhibited by oxygen. Here lies a paradox: The process of nitrogen fixation is extremely energy demanding and as such requires aerobic respiration, yet the enzyme can only function when oxygen is restricted. Throughout evolution, bacteria have found inventive ways of solving this paradox, one of which is to associate with plants that supply the energy required for the reaction and create an oxygen-regulated environment. Legumes are one such group of plants that form specialised root nodules, whose cells are packed full of internalised bacteria, living in an environment optimised for nitrogen fixation. In this root nodule symbiosis (RNS), the plant delivers carbohydrates derived from photosynthesis, while the bacteria fulfil the totality of the plants’ nitrogen needs, creating benefits for both partners.

The RNS already contributes significantly to sustainable food production: We and our domestic animals consume billions of tonnes of legume seeds, produced without nitrogenous fertilisers. However, the existence of the RNS has inspired scientists for many decades as a route to deliver free, sustainable, and low-polluting sources of reactive nitrogen to a much broader array of crops, especially our staple cereal crops, which are hungry fertiliser consumers. There are multiple possible biological routes to solving the nitrogen problem in agriculture, and these are extremely active areas of current research: enhancing the utility of free-living diazotrophic interactions with cereal roots [10,11]; engineering the bacterial nitrogenase enzyme directly into plant cells [12–14]; and transferring the RNS from legumes to cereal crops. This latter approach has a prototype in the legumes that has already delivered the totality of the plants’ nitrogen needs, and it is here where we focus our attention in this article. The last few decades have seen major advancements in our understanding of the RNS that we argue brings within reach a sustainable and equitable solution for global food production. The world’s population continues to expand and will do so over a period of profound global climate change. Therefore, we need this solution now more than ever, and we will attempt to outline the extent of our knowledge in the RNS and the gaps that might limit the development of this technology over the following decades.

### The root nodule symbiosis

Diazotrophic bacteria that form symbioses with legumes are referred to as rhizobia, and their presence in soils is widespread across the planet. Establishing the symbiotic relationship requires the plant root and bacteria to find each other in the soil, involving a molecular dialogue between host plant roots and rhizobia, which, in turn, induces host and bacterial processes associated with bacterial infection and nodule organogenesis. We suggest four principal steps in this process that usefully define the critical areas to consider when striving to transfer nitrogen fixation: signal perception, bacterial infection, nodule organogenesis, and the establishment of the environment for nitrogen fixation. How nodulation has evolved remains an ongoing discussion, but the latest work proposes a single origin in the evolution of nodulation, followed by multiple losses [15,16]. Associated with the multiple losses has been a consistent loss of only a very few genes: the Nod factor (NF) receptor *NFP* associated with rhizobial recognition; the master regulator of the root nodule symbiosis *NIN* and *RPG*, which is required for bacterial infection. This highlights a theme that runs throughout this review: Evolution of nodulation is not associated with massive increases in new genetic components; rather, a few key regulators allow novel networking of preexisting processes. These key regulators, which may be defined by those genes lost with loss of nodulation, match the processes we outline below.

## I. How do plants perceive nitrogen-fixing bacteria?

**Fig 1:**
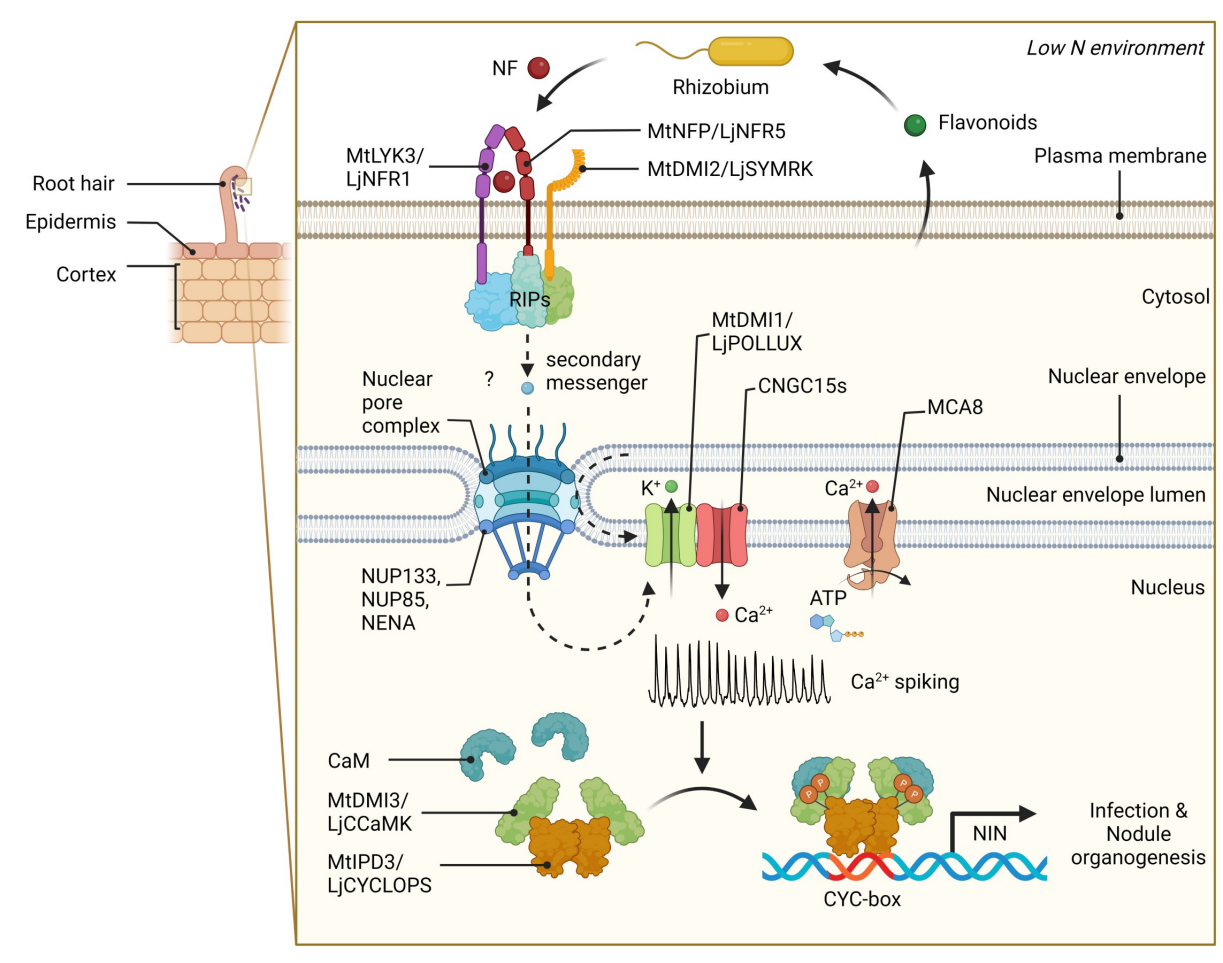
NF perception and the common symbiosis signalling pathway. Flavonoid exudates from legume roots act as signals to relevant rhizobia in the soil, activating production of NF. A receptor complex at the root surface allows NF recognition, through binding to LysM receptor kinases LYK3 (also known as NFR1) and NFP (also known as NFR5), coupled to the LRR-containing receptor kinase SYMRK (also known as DMI2) that activates downstream signalling. A number of RIPs have been identified that may facilitate downstream signal transduction including ROP-GTPases and GEFs, which are particularly associated with rhizobial infection; a group of receptor-like cytoplasmic kinases, which includes SYMRK INTERACTING PROTEINS and the NFR5-INTERACTING CYTOPLASMIC KINASE4 that can activate phosphorylation cascades; and a HMGR, which interacts with SYMRK and induces the production of mevalonate. The action of one or many of these RIPs may produce a secondary messenger that links receptor activation at the plasma membrane to induction of cation channels on the nuclear envelope. The cyclic nucleotide-gated channels CNGC15s release calcium from the nuclear envelope lumen into the nucleus, while other cation channels are required for symbiotic calcium spiking, CASTOR and POLLUX (also known as DMI1): DMI1 interacts with CNGC15 and appears to coordinate the release of calcium from this channel. The calcium ATPase MCA8 pumps calcium back into the nuclear envelope to maintain nuclear calcium homeostasis. Components of the nuclear pore complex, like NUP85, NUP133, and NENA, are also required for symbiosis signalling, and these are thought to direct the necessary channels onto the inner nuclear envelope. The coordinated action of the channels and pumps creates nuclear calcium oscillations that promote the CCaMK (also known as DMI3). CCamK/DMI3 phosphorylates CYCLOPS/IPD3, which, in turn, promotes symbiotic transcription, such as the induction of *NIN*. Gain-of-functions in *NFR1*, *NFR5*, *SYMRK*, *DMI1*, *CCaMK*, and *CYCLOPs*, all autoactivate nodulation, demonstrating that activation at any point in this pathway is necessary and sufficient for nodule initiation. Created with BioRender.com. CaM, calmodulin; CCaMK, calcium and CaM-dependent serine/threonine protein kinase; DMI2, DOES NOT MAKE INFECTIONS 2; GEF, Guanine-nucleotide Exchange Factor; HMGR, 3-hydroxy-3-methylglutaryl-CoA reductase; IPD3, INTERACTING PROTEIN OF DMI3; Lj, *Lotus japonicus*; LysM, lysine motif; Mt, *Medicago truncatula*; NF, Nod factor; NIN, NODULE INCEPTION; NUP, NUCLEAR PORE COM-PLEX PROTEIN; RIP, receptor-interacting protein; ROP-GTPase, Rho of plants–guanosine triphosphatase.

The dialogue between legumes and rhizobia is initiated by the secretion of (iso)flavonoids and betaines from the legume roots to attract rhizobia and activate the transcription of rhizobial nodulation (nod) genes, involved in the biosynthesis of NFs [17]. NFs are decorated lipochitooligosaccharides (LCOs) [18], which serve as rhizobial signals to the host plant and are recognised by a receptor complex at the root surface, principally made of lysine motif (LysM)-containing receptor-like kinases and a leucine-rich repeat receptor-like kinase [19–21] (Fig 1). Receptor activation triggers the common symbiosis signalling pathway, shown to be active in root epidermal cells at the point of bacterial recognition [22] (Fig 1). Central to symbiosis signalling is the activation of calcium oscillations within the nucleus and nuclear-associated cytoplasm that is coordinated by an array of cation channels located on the nuclear membranes [23–25]. It remains unclear precisely how receptor activation at the plasma membrane is able to promote calcium oscillations; however, a suite of receptor-interacting proteins have been identified that may act to induce secondary messengers to promote the channels on the nuclear membranes [26] (Fig 1). The nuclear calcium oscillations are decoded by a calcium signalling complex made up of a calcium and calmodulin-dependent protein kinase (CCaMK) [27,28], coupled to a transcription factor (CYCLOPS) [29,30], phosphorylation of which promotes symbiosis-associated gene expression (Fig 1).

Symbiosis signalling is not limited to legumes; rather, this signalling pathway appeared at the dawn of plant evolution and has been used repeatedly across the plant kingdom to facilitate intracellular interactions with beneficial microorganisms [31]. Notably among such interactions is that between plants and arbuscular mycorrhizal fungi (Box 1), which appears to be the earliest of all beneficial microbial associations with plants and the founding interaction that facilitated the evolution of symbiosis signalling [32]. Considering this early emergence in the evolution of plants, symbiosis signalling is present in most plant species, in particular, our most important cereal crops: rice, wheat, and maize [33–35]. The utilisation of this signalling pathway for recognition of rhizobial bacteria in legumes does not appear to be associated with a change in the nature of the signalling pathway, since homologous genes in cereals can complement mutants in their legume counterparts, allowing interactions with nitrogen-fixing rhizobia [36–38]. What does appear to have changed is the stringency of signal recognition through the receptor complex that, in legumes, allows a very stringent perception of specifically decorated LCOs produced by their rhizobial symbiont [35]. In contrast, cereals show little to no discrepancy for decorations around the LCO backbone and show equal activation of symbiosis signalling by simple chitooligosaccharides, whose receptor CERK1 appears to play an important role, alongside the LCO receptors, for initiating the arbuscular mycorrhizal symbiosis [33,34,39,40]. The presence of symbiosis signalling in cereals provides an excellent foundation for engineering cereal crops for associations with nitrogen-fixing bacteria, with engineering focusing on the stringent recognition of decorated LCOs produced by rhizobia and the specific mode of downstream activation of cellular and developmental processes associated with accommodating nitrogen-fixing bacteria.

**Box 1. Arbuscular mycorrhizal fungi**

**Arbuscular mycorrhizal fungi** are mutualistic symbiotic soil microorganisms that colonise the roots of most land plants. The fungi increase nutrient availability for their host plants and are especially important for the uptake of phosphate and nitrogen, but they also provide water and micronutrients. Mycorrhizal fungi invade the root through epidermal cells and intracellularly colonise root cortical cells, establishing highly branched hyphae, known as **arbuscules**, which provide an interface for nutrient exchange between the plant and the fungus. In return for these nutrient services, the plant provides all of the carbon the fungus needs, mostly in the form of lipids derived from photosynthetic carbon capture.

### Unsolved mystery 1: What are the transduction mechanisms that allow a rhizobium-specific output when activating the generic symbiosis signalling pathway?

Legumes appear to have evolved very stringent recognition of rhizobial-produced LCOs, which likely emerged following a whole genome duplication and further expansion of the LysM receptor-like kinase class [41]. Recent work has demonstrated the precise residues in the legume receptors that not only define an LCO-receptor, but also allow stringent recognition of specifically decorated LCOs [42], providing the framework for engineering such receptors in cereals. Activation of symbiosis signalling in legumes is sufficient to induce nodulation [27,28,43,44], something that does not appear to happen in cereals. Mycorrhizal activation of symbiosis signalling induces the expression of a mycorrhizal-specific transcription factor, *REDUCED ARBUSCULAR MYCORRHIZA1* (*RAM1*), through the action of a transcriptional complex made up of CCaMK-CYCLOPS-DELLA proteins [45]. Similarly, the expression of the rhizobial-specific transcription factor *NODULE INCEPTION* (*NIN*) [30] is also activated through a similar CCaMK-CYCLOPS-DELLA complex [45]. How do legumes utilise the same signalling to promote quite different transcriptional outputs, and how is this specificity linked to the precise recognition of nitrogen-fixing bacteria? This question sits at the heart of utilising the preexisting signalling capabilities present in cereals, to allow the engineering of rhizobial recognition and the regulation of the relevant cellular and developmental processes to allow accommodation of bacteria in engineered cereals.

### Unsolved mystery 2: How does the NF-ndependent symbiosis work?

The perception of NFs plays an essential role in selecting the suitable symbiont for the legume host. However, some rhizobia can bypass this process and induce nodulation in a discrete number of legume species, in an NF-independent manner [46–51]. This does not negate symbiosis signalling; rather, an alternative mechanism to activate this signalling pathway appears to exist, and at least in one situation, this is the function of a bacterial effector delivered through the type III secretion system [51,52]. Understanding this NF-independent mechanism for activation of symbiosis signalling could provide an alternative approach to engineering the receptors in cereals to drive appropriate induction of symbiosis signalling, upon rhizobial contact.

## II. How do plants control the bacterial infection process?

**Fig 2:**
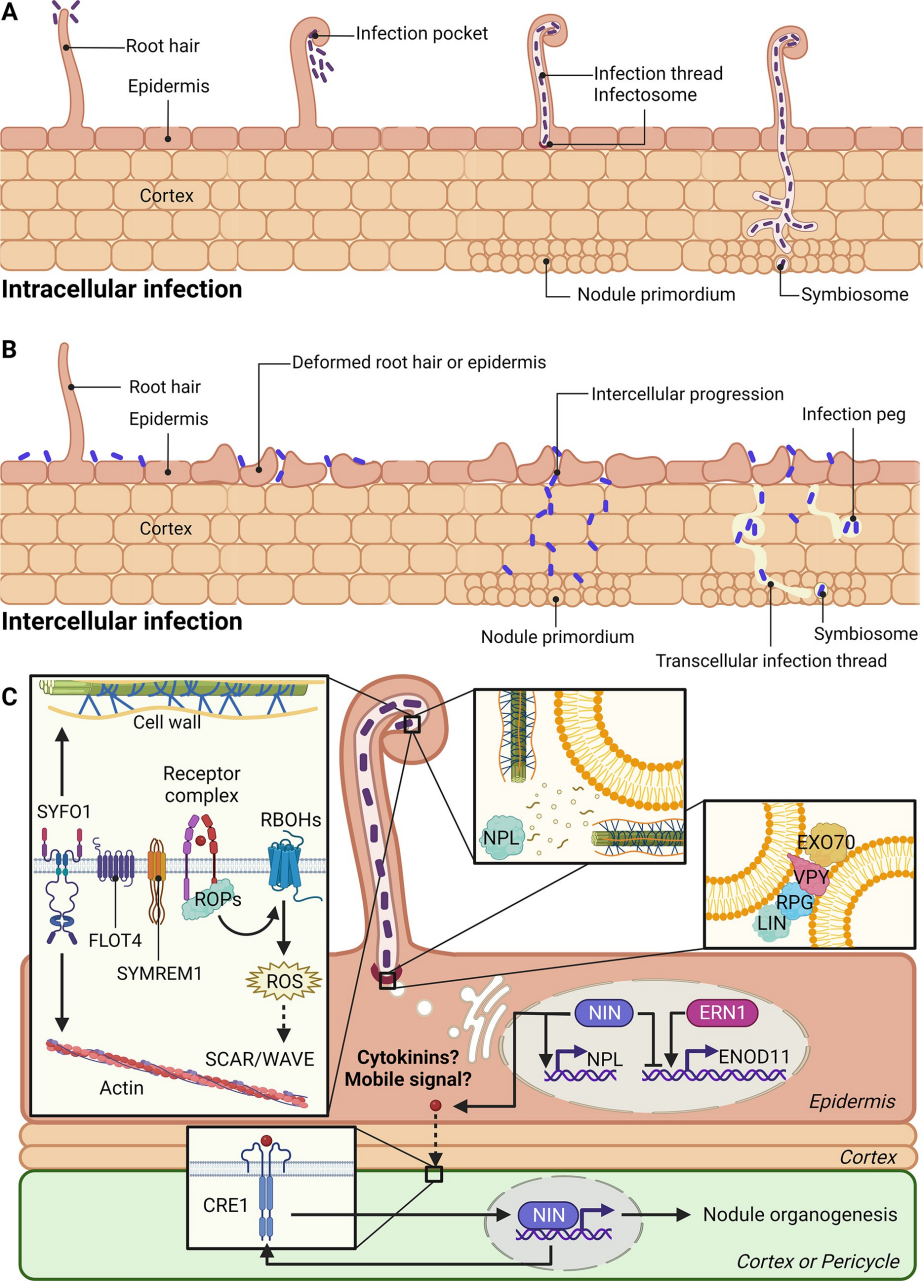
Rhizobial infection. Rhizobia can enter the root immediately through a process of intracellular infection (**A**) or through differing levels of intercellular infection (**B**). Whichever route is taken, bacteria always end up intracellularly accommodated. (**C**) Initiation of intracellular accommodation starts with receptor activation through the stringent perception of NFs or exopolysaccharides. ROPs, which interact with the NF receptors, activate RBOHs, which regulate reactive oxygen species, which can coordinate multiple aspects of cell functionality and signalling. The SCAR/WAVE complex, which governs the ARP2/3 complex coordinating actin dynamics for infection thread initiation. Actin dynamics alongside two scaffold proteins, FLOT4 and the remorin protein SYMREM1, facilitate the formation of a nanodomain and reduce the mobility of the NF receptors, a process vital for rhizobial infection. Coordinating the microtubule organisation with cell wall and plasma membrane dynamics is in part fulfilled by SYFO1. NPL, which plays a critical role in cell wall remodelling for infection thread development, is stimulated and accumulates in the infection pocket in response to NFs. The infectosome, which is made up of VPY, the E3 ligase LIN/CERBERUS, RPG, and the exocyst complex EXOCYST subunit H4 (EXO70 H4), is located at the tip of infection threads and regulates polar development by controlling vesicle membrane fusion. Perfect synchronisation of infection and nodule production is required for effective nodulation, which is regulated by NIN and ERN1. NIN regulates rhizobial infection in epidermal cells by up-regulating NPL. On the other hand, in epidermal cells, NIN competes with ERN1 to limit the production of ENOD11 and probably other genes. At the same time, NIN promotes the transfer of an unknown mobile signal, perhaps cytokinin, from the epidermis to the cortex to initiate cell divisions in cortical cells, leading to the formation of the nodule meristem. Created with BioRender.com. ARP2/3, actin-related protein 2/3; ENOD11, EARLY NODULIN 11; ERN1, Ethylene Response Factor Required for Nodulation 1; FLOT4, FLOTILLIN 4; LIN, LUMPY INFECTION; NF, Nod factor; NIN, NODULE INCEPTION; NPL, NODULE PECTATE LYASE; RBOH, Respiratory Burst Oxidase Homolog; ROP, Rho of plants; RPG, RHIZOBIUM-DIRECTED POLAR GROWTH; SCAR/WAVE, Suppressor of cAMP receptor defect/WASP family verpolin homologous protein; SYFO1, SYMBIOTIC FORMIN 1; VPY, VAPYRIN.

NF signalling sets in motion two processes: the activation of nodule primordia in inner root tissues and the entrapment of rhizobial bacteria at the root surface, with the initiation of an infection process that delivers bacteria to the developing nodule. The initiation of both infection and nodulation is controlled by the master regulator *NIN* [22,53]. In all cases, the association results in intracellular bacteria inside the cells of the nodule. However, the routes to this can be either through intercellular infection, i.e., bacteria dividing in the intercellular spaces of the root or intracellular infection through tubular invaginations of the root cells called infection threads (Fig 2A and 2B). The principal mode of infection in the legume genetic model species is intracellular; thus, this is the area where most knowledge currently exists. However, recent discoveries are beginning to give insights into the genetic components that underpin intercellular infection, and we cover both below.

Analogous to what has been shown for symbiosis signalling, some of the plant components associated with the bacterial infection process are derived from the association with arbuscular mycorrhizal fungi, in particular, the processes associated with the extension of the infection thread that is closely analogous to a fungal hypha invading a plant cell. However, whereas fungi have the ability to create a closed compartment at the surface of the plant that allows them to create a pressurised system to push against the turgor of the plant cell [54], bacteria lack this ability, meaning the initiation steps of the infection thread appear quite different to fungal infection mechanisms, and the commonalities occur only at the stage of the progression of the infection structures. From the perspective of engineering RNS into cereals, the key is to differentiate the novelty in the bacterial infection mechanisms compared to the conserved processes already present to support the arbuscular mycorrhizal symbiosis.

### Intracellular infection

The perception of NFs induces root hair tip growth reinitiation and curling, which encloses rhizobia and forms an infection chamber, also known as an infection pocket. The cell wall surrounding the infection chamber is degraded, which allows rhizobia to enter the root hair cell via plasma membrane invaginations. The continuous fusion of membrane vesicles at the infection site extends the membrane invagination and forms an intracellular tube, named the infection thread. The infection thread continuously elongates and branches through the root cortex, growing towards the nodule primordium. Once the infection thread reaches the nodule, “infection droplets” containing rhizobia are released into the nodule cells, resulting in membrane-bound symbiosomes, the organelle-like structures where nitrogen fixation occurs (Fig 2A).

Facilitating intracellular bacterial infection carries risks for the host plant because multiple different rhizobia present in the soil might also enter the plant tissues, including poor nitrogen fixers or potential pathogens. Therefore, mechanisms of selecting efficient symbiotic nitrogen-fixing bacteria strains are required for host plants to overcome this dilemma. During root hair infection, generally, single bacteria are entrapped, creating a clonal population of bacteria within the infection thread [55]. Subsequently, host plants can also apply conditional sanctions to inhibit the growth of underperforming nodules that are colonised by ineffective nitrogen fixers [56]. Notably, all these processes require the host plant to have the ability to recognise and distinguish specific rhizobia. Therefore, additional levels of stringency are attached to the recognition of bacteria before the infection is activated. Multiple studies demonstrate the importance of NF perception for the initiation of infection threads, and in some species, this stage requires a much higher degree of specificity for NF recognition [41,57]. Alternatively/in addition, other bacterial signals appear to play a role, with plant perception of exopolysaccharides on the surface of the rhizobia providing an additional level of stringency in bacterial recognition at this infection stage [58]. Two scaffold proteins, FLOTILLIN 4 (FLOT4) and the remorin protein SYMREM1, facilitate the formation of membrane nanodomains, reducing the mobility of the NF receptors, and this appears vital for appropriate rhizobial infection [59]. While the symbiosis signalling pathway is important for infection, there is little evidence that this transduction pathway is the primary link between receptor activation and the initiation of infection threads. Rather, a single calcium transient across the plasma membrane [60] that correlates with a burst of reactive oxygen species [61–63], appears associated with infection. Receptor-associated ROPs [64,65] activate Respiratory Burst Oxidase Homologs (RBOHs), and this is a likely mechanism for provoking cytoskeletal remodelling [66], initiating the infection thread [67] and regulating the dynamics and assembly of actin needed to redirect root hair polar growth [66,68]. ROPs also modulate actin dynamics via the SCAR/WAVE (Suppressor of cAMP receptor defect/WASP family verpolin homologous protein) complex [69–71], which allows actin nucleation, required for infection thread initiation [71–75] (details in Fig 2). Translating an activated microdomain of receptors at the site of bacterial attachment into an invagination of the plasma membrane requires physical changes to the membrane, and the first indicators of how this might happen has come with the description of a *SYMBIOTIC FORMIN 1* (*SYFO1*) that provides a cell wall-plasma membrane-cytoskeleton continuum [76] (Fig 2) that can perhaps provide the initial scaffold to invaginate the membrane.

A mature cell wall is a barrier to the redirection of polar cell growth, and to achieve cell wall remodelling, the plasticity of the cell wall needs to be altered. Precisely, localised cell wall degradation at the site of the infection pocket is facilitated by directed secretion of NODULE PECTATE LYASE (NPL), which is transcriptionally induced by NFs and accumulates at the site of the infection pocket [53,77]. NPL degrades pectin, reinstating plasticity to the cell wall, which, in part, allows the formation of new sites for polar growth [78,79]. Analogous to an infection thread is the invasion of plant cells by mycorrhizal fungal hyphae. It has been demonstrated that during the formation of an arbuscule (Box 1), all vesicle transport is redirected to the sites of fungal penetration. As such, any secreted protein that is expressed during this time is targeted to the peri-arbuscular space [80]. Although not proven, an analogous situation could easily explain the unique proteins that are delivered to the growing bacterial infection thread: Any protein expressed at a time of infection thread growth, such as NPL, will, by default, be targeted to the tip of the growing infection thread [79].

Once initiated, progression of the infection thread appears to function in a manner analogous to the invasion of the plant cell by mycorrhizal fungal hyphae. Similar cellular structures have been reported during colonisation by both organisms [81,82], with the nucleus lining up to the site of infection and cytoplasmic bridges becoming apparent between the infection site and the nucleus, predicting the path of the infection structures. Beyond these commonalities in cellular structures are also commonalities in the genetic components or homologous genetic components required for the extension of both mycorrhizal fungal and rhizobial infection structures. These genetic components make up a protein complex, the infectosome, which forms at the tip of infection threads regulating exocytosis and is composed of VAPYRIN (VPY), the E3 ligase LUMPY INFECTION (LIN)/CERBERUS, and EXOCYST subunit H4 (EXO70 H4) [83–85]. The infectosome governs polar growth of infection threads by regulating the fusion of vesicles (Fig 2). In addition, the coiled-coil protein RHIZOBIUM-DIRECTED POLAR GROWTH (RPG) [15,86], which is essential for rhizobial infection, is located at both the perinuclear cytoplasm and the tip of the growing infection thread [79], providing a tantalising link to the nucleus that appears to guide the growth of the infection thread. Note that *RPG* is one of the few genes shown to be lost with the loss of nodulation, highlighting the central role it appears to play in allowing the existence of the RNS [15,16].

### Intercellular infection

Intercellular rhizobial infection is observed in approximately 25% of legume species [87,88]. Here, the rhizobia enter the root tissue by degrading the middle lamella and primary cell wall and progressing through the intercellular space between epidermal and cortical cells (Fig 2B). In some interactions, rhizobia proliferate in the intercellular spaces and only become intracellular at the point of colonising the cells of the nodule [89,90]. In other cases, rhizobia induce cell death to form intercellular infection pockets in dead or collapsed cells, from which intracellular infection pegs or threads are initiated [89,90].

### Unsolved mystery 3: What genetic adaptations allowed rhizobia to infect plant cells intercellularly?

Intercellular infection appears simpler than intracellular infection through infection threads and thus is an attractive target for engineering, when considering transferring RNS to other crops. Since intercellular infection has been observed in the nitrogen-fixing lineages across the nitrogen-fixing clade: Fabales; Fagales; Cucurbitales; and Rosales, intercellular infection is considered a more ancient pathway for flowering plants that established a nitrogen-fixing symbiosis with bacteria [90], which likely evolved from intercellular diazotrophic endophytes [91,92]. Interestingly, recent studies have shown that Fabales and Fagales are sister clades [93,94], and the root hair–based intracellular infection likely evolved before their diversification because several reports indicate that the basal clades in Fabaceae [95,96] and several nodulating actinorhizal lineages in Fagales utilise intracellular infection mechanisms [97,98]. Therefore, the most recent common ancestor of Fabales and Fagales that accepted rhizobium as a microsymbiont might already have had the genetic framework to allow root hair–based intracellular infection [99]. Consequently, intercellular infection observed within legume lineages is currently considered a derived trait in Fabales. Most of these host plants in Cucurbitales and Rosales that show such alternative infection strategies are challenging genetic systems, making understanding this intercellular infection mode difficult. However, recent advances in studying species in Fabales and Fagales are changing this situation. The discovery of situations whereby the model legume *Lotus japonicus* undergoes intercellular rather than intracellular infection [100] has allowed a genetic dissection of this infection route [101], and the development of *Aeschynomene evenia* as a genetic model is also allowing the dissection of alternative means of rhizobial infection [48,49]. To date, these studies have only shown the importance of genetic components already known to be important for intracellular infection, but these platforms should soon allow novel genetic components to be discovered with specific functions in intercellular infection. Compared with intracellular infection, our understanding of intercellular infection is still relatively limited. It remains unclear whether intercellular infection will be easier to engineer, although it logically appears that way. Further studies and knowledge of these intercellular colonisation systems could provide a potential alternative path for crop engineering. This information might be precious, especially when our engineering target crop species are monocots, like maize, rice, and wheat, which are distantly related to the nitrogen-fixing clade of plants.

## III. How do plants regulate nodule organogenesis?

**Fig 3:**
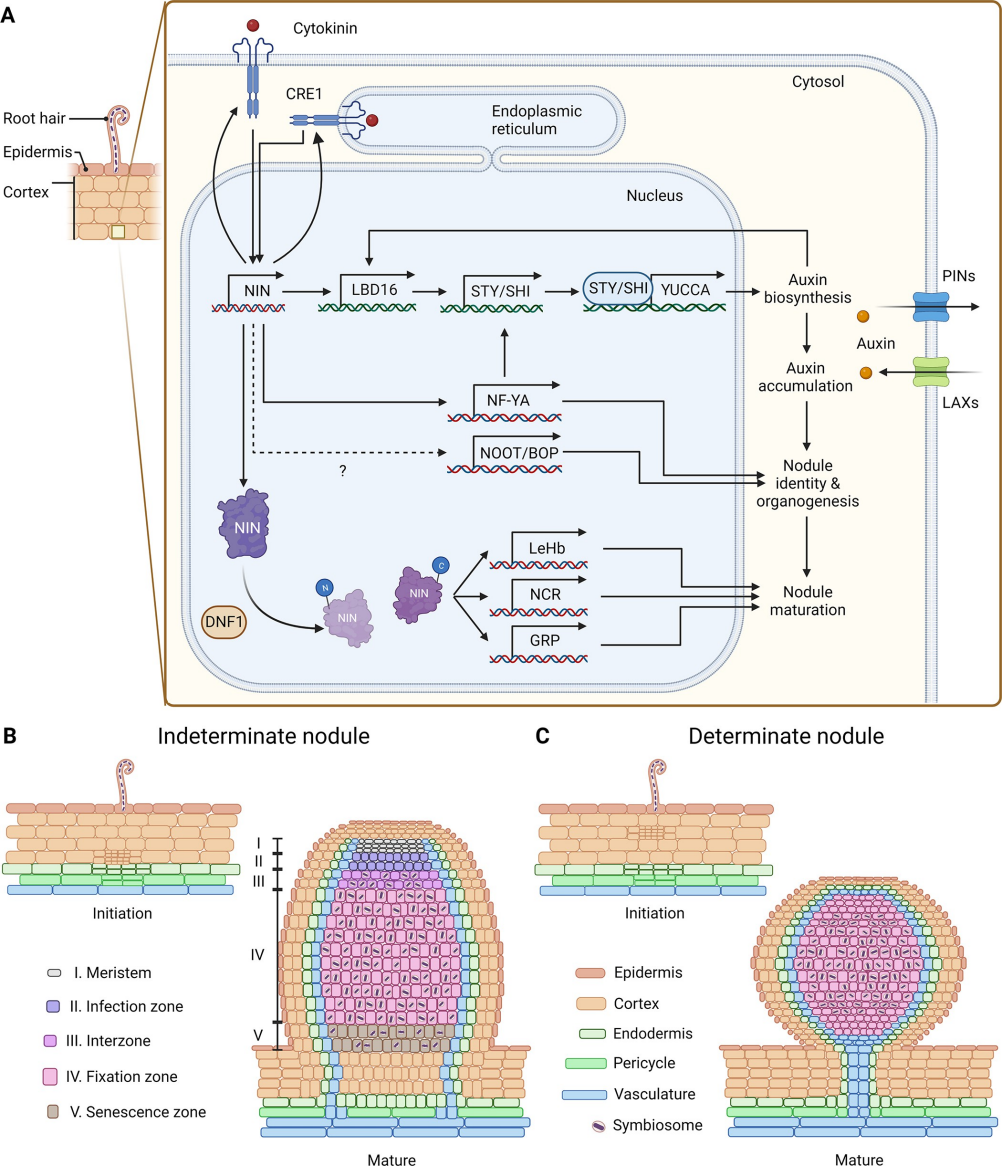
Nodule organogenesis. (**A**) The regulatory network underpinning nodule development. In cortical cells, activation of the cytokinin receptor CRE1/LHK1 induces *NIN* expression. In return, NIN promotes the transcription of *CRE1*, creating a feedforward loop that can increase cytokinin signalling and NIN accumulation. NIN controls the expression of *LBD16*, an auxin-responsive transcription factor that activates the auxin symbiosis pathway via *SHI/STY* transcription factors, which, in turn, promote expression of *YUCCAs*, rate-limiting enzymes in auxin biosynthesis. The expression and distribution of auxin transporters, PINs and LAXs, are precisely regulated during different stages of nodule development to control the dynamics of the accumulating auxin maximum. NIN also induces *NF-YA1* expression, which further enhances *STY/SHI* expression, as well as likely contributing to other aspects of the nodule meristem and bacterial infection. We propose the existence of an unknown component that dictates nodule identity, which, in turn, likely affects expression of *NOOT* genes that are necessary to maintain nodule identity. NIN also controls the nodule maturation process to transition into the nitrogen-fixing state. The DNF1 signal peptidase complex cleaves the NIN protein and generates a C-terminal NIN fragment, which activates genes involved in bacteroid differentiation and nitrogen fixation, including NCRs, GRPs, and leghemoglobin. (**B**, **C**) The developmental patterns of indeterminate and determinate legume nodules at initiation and maturation stages. (**B**) For indeterminate nodules, the initial cell divisions forming the nodule primordia occur in inner cortical cells, while in determinate nodules, this occurs in the outer cortical cells (**C**). Despite their anatomical differences, cell divisions in the pericycle have been observed in both nodule types. (**B**) Mature indeterminate nodules contain a persistent meristem at the tip of the nodule, which is commonly observed in *M*. *truncatula* and *Pisum sativum*. (**C**) Mature determinate nodules form without having a persistent meristem, which is often seen in *L*. *japonicus*, *Phaseolus vulgaris*, and *Glycine max*. Created with BioRender.com. DNF1, DEFECTIVE IN NITROGEN FIXATION1; GRP, glycine-rich peptide; *LBD16*, *LATERAL ORGAN BOUNDARIES DOMAIN 16*; NCR, nodule-specific cysteine-rich peptides; NIN, NODULE INCEPTION; *NOOT*, *NODULE ROOT*; *SHI/STY*, *SHORT-INTERNODES/STYLISH*.

A nodule must form below the site where rhizobia make contact with the root surface, and as such, unlike lateral roots, nodule development cannot be predisposed to a discrete group of cells. Rather, a nodule initiation has to occur de novo, in cortical and pericycle cells that are fully differentiated and ordinarily would not undergo further cell divisions. The site of nodule initiation, whether from the outer or inner root cortex, differs between legume species and is associated with two nodule types, indeterminate and determinate, which differ in the maintenance of an apical meristem (Fig 3) [102–105]. In all situations, the transcription factor NIN is the master regulator of integrating the developmental processes necessary to accommodate nitrogen-fixing bacteria: activating infection threads in epidermal cells and simultaneously triggering nodule primordia formation in the root cortex. NIN promotes an unknown mobile signal, potentially cytokinin, which links the developmental changes in the epidermis to the root cortex [22]. Cytokinin and *NIN* form a feedforward loop in the root cortex, with cytokinin signalling activating *NIN* expression [106], and NIN activating expression of the nodulation-associated cytokinin receptor *CRE1* [22] (Figs 2C, 3A). This creates a signalling centre within a discrete group of cortical cells, which is sufficient to promote nodule organogenesis [107]. Such cytokinin induction of a root organogenesis program appears unique to legumes; indeed, in other plant species, the presence of cytokinin suppresses root organogenesis [107].

### Unsolved mystery 4: What is the signal moving from the epidermis to the cortex and pericycle to activate cytokinin signalling and nodule primordium formation?

Nodule development must be temporally and spatially coordinated with bacterial infection, and underpinning this coordination is cell-to-cell communication linking activation of the symbiosis signalling pathway in the root epidermis to the promotion of cell divisions in the root cortex and pericycle [22,108]. NFs themselves seem unable to diffuse into the deeper cortical cell layers [109], and evidence supports the notion that a product of *NIN* in the epidermis may act as the mobile signal [22]. This could be cytokinin, and, indeed, genes involved in cytokinin biosynthesis and accumulation are induced in epidermal cells upon NF perception [110,111] (Fig 2C). However, recent work has demonstrated that infection by mycorrhizal fungi and treatments with elicitors from mycorrhizal fungi promote cell divisions in the cortex and the emergence of lateral roots in both legumes and cereals [81,112]. This suggests that the cell-to-cell communication linking symbiosis signalling at the root surface to the induction of root organogenesis may be another mechanism in nodulation derived from the more ancient mycorrhizal symbiosis. If correct, this places the novelty for nodulation being defined by the ability to amplify this signal and drive a novel aspect of development. Whether cytokinin or a component derived from the mycorrhizal symbiosis, the nature of this diffusible signal linking root tissues during nodule development, is both interesting and strategically important.

The unique ability of legumes to respond to cytokinin with the promotion of nodule initiation appears to be defined by the cytokinin regulation of *NIN*, which, in turn, recruits *LATERAL ORGAN BOUNDARIES DOMAIN 16* (*LBD16*), a central regulator of lateral root development, to facilitate nodule organogenesis [113,114]. LBD16 coordinates the accumulation of a local auxin maximum, through the regulated expression of *SHORT-INTERNODES/STYLISH* (*SHI/STY*) transcription factors that, in turn, initiate expression of the YUCCAs, rate-limiting enzymes involved in auxin biosynthesis [113]. NIN also promotes the expression of *Nuclear Factor-Y* (*NF-Y*) subunit genes, such as *NF-YA1* and *NF-YB1* [115–121], which, in both plants and animals, play vital roles in the regulation of the cell cycle and cell proliferation [122,123]. These genes have also been demonstrated to further promote auxin biosynthesis during nodulation via up-regulation of *SHI/STY* [116,117,119] (Fig 3). The local accumulation of auxin at the site of nodule formation is further facilitated by the suppression of polar auxin transport [124,125], allowing accumulation of auxin within the local site of *NIN* induction (Fig 3). Auxin accumulation appears to be further promoted by auxin activation of *LBD16* expression, creating a feedforward loop. These multiple avenues for *NIN* regulation of auxin accumulation allow the amplification of a local cytokinin signal into an auxin “hub,” creating a new developmental centre for de novo meristem formation.

While initiation of a nodule converges on multiple aspects of lateral root development [113,114], what emerges from a nodule meristem is quite different to a lateral root; in particular, nodules of legumes possess peripheral vascular strands and contain many cells able to harbour intracellular bacteria. The demonstration of a single origin for the evolution of nodules [15,16] suggests that the structure of nodules in non-legumes, so called actinorhizal species, may represent the primitive state of nodules: Such nodules possess a centralised vasculature [97,126], implying that the peripheral vasculature observed in legume nodules, probably evolved from an intermediary structure more analogous to a lateral root. This, alongside the genetic dissection of nodulation, suggests that lateral root development underpins the formation of a nodule, but this dictates the need for developmental regulators that impart a nodule identity onto the cells of the nodule primordia. *NODULE ROOTs* (*NOOTs*), orthologs of the *BLADE-ON-PETIOLE1/2* (*AtBOP1/2*) genes encoding ankyrin repeat and BTB/POZ domain-containing cotranscriptional regulators in *A*. *thaliana*, are essential for maintaining nodule identity, after initiation: In their absence, nodules revert back to a lateral root [127,128] (Fig 3), with lateral roots emerging from nodules of *noot* mutants. This implies that *NOOTs* are required to maintain the identity of the nodule; however, the regulators that initially drive this nodule identity have yet to be defined.

### Unsolved mystery 5: What is the mechanism that dictates nodule identity?

Several reports have indicated that rice and *Brachypodium* can initiate nodule-like structures upon auxin treatment [129–131] but do not do so following treatments with cytokinins [132]. This supports the notion that *NIN* induction, as a function of cytokinin [106], is one of the key aspects of novelty within legumes. *NIN* promotes the initiation of a meristem, with many parallels to a lateral root, yet what emerges is developmentally quite different. We do not yet know the regulators that allow specific aspects of nodule development to emerge from a lateral root meristemic program. NOOTs are clearly a component of this nodule identity, but their principal function appears to be maintaining nodule identity, rather than activating it. There must be a developmental regulator, likely promoted by *NIN*, which acts in parallel to *LBD16*, to drive the novel development we see in a nodule. This novel regulator likely controls the expression of *NOOTs*. Understanding these regulator(s) is central to our ability to engineer this novel mode of root development into our target cereal crops.

## IV. How do plants create an appropriate environment for nitrogen- fixation?

**Fig 4:**
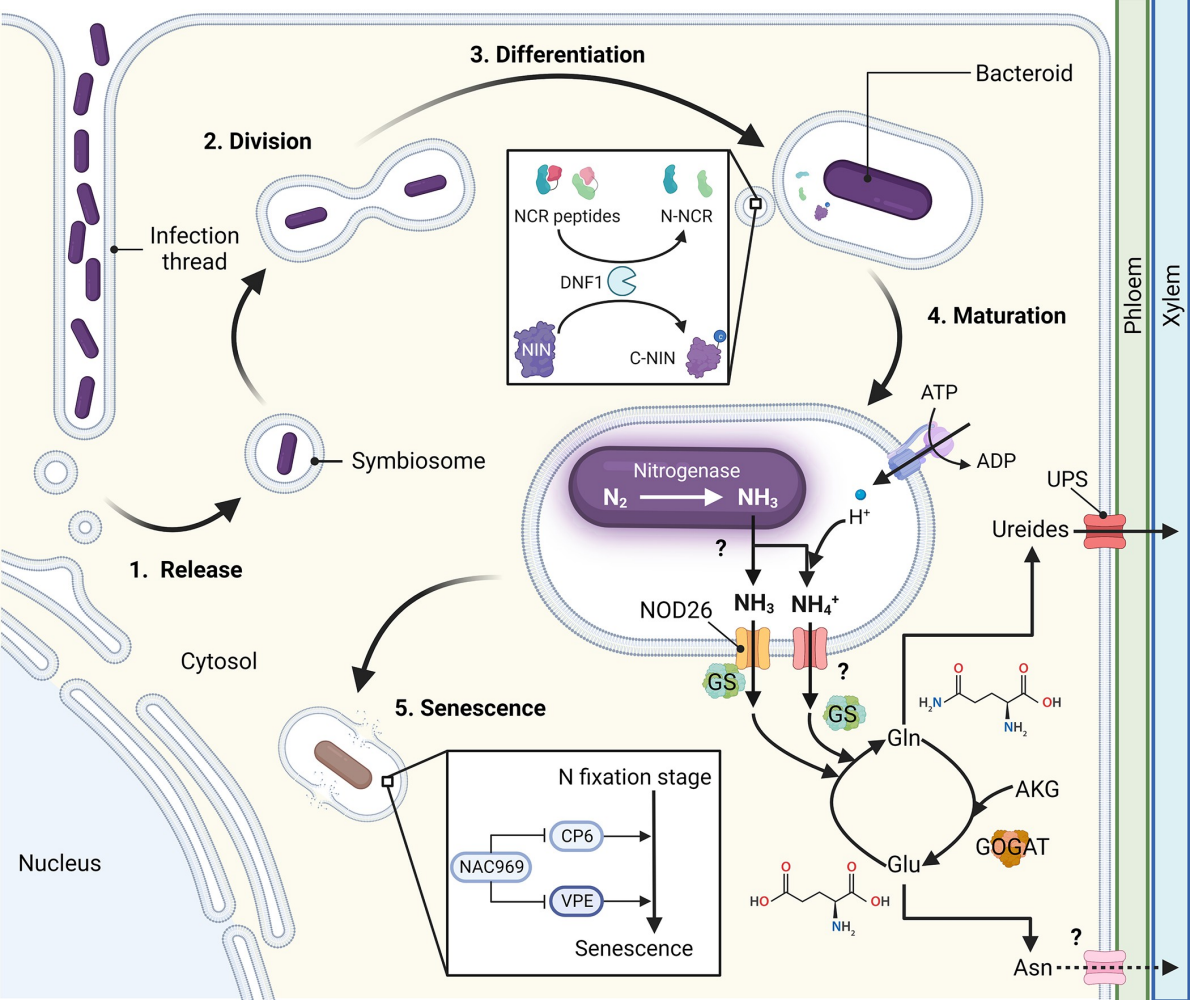
Creating an environment for nitrogen fixation and nitrogen delivery. Once the infection thread reaches the nodule primordium, infection threads release droplets into the cell containing bacteria, always surrounded by a plant-derived membrane. These structures, the so-called symbiosomes, are organelle-like. Symbiosomes can continue to divide, and the bacteria can differentiate into bacteroids. The DNF1-cleaved N-NCR is delivered into the symbiosome via membrane vesicle trafficking and induces bacteroid differentiation. DNF1 also proteolytically cleaves the NIN protein and generates a C-NIN, activating genes involved in terminal differentiation and nitrogen fixation. The nitrogenase enzyme complex in rhizobia converts N_2_ into ammonia that is released from the bacteroid via diffusion or unknown channels. The H+-ATPases on the symbiosome membrane create an acidic peribacteroid space, which traps ammonium by protonating ammonia and producing ammonium cations. Ammonium cations are exported into the cytoplasm of plant cells and then assimilated into Gln and Glu by GS and by GOGAT, transferring the amide group from Gln to AKG. Depending on legume species and nodule types, Gln and Glu are converted into Asn or ureides for long-distance transport through the xylem. In the end, the nodule undergoes senescence and bacteroids lyse. Different CPs are highly expressed in nodules at the senescence stage, especially a papain peptidase (CP6) and a VPE, which are controlled by a transcription factor NAC969. Created with BioRender.com. AKG, α-ketoglutarate; Asn, Asparagine; C-NIN, C-terminal NIN peptide fragment; CP, cysteine proteinase; Gln, glutamine; Glu, glutamate; GOGAT, glutamate synthase; GS, glutamine synthetase; NAC, NAM/ATAF/CUC; NAC969, NAC-encoding 969; NIN, NODULE INCEPTION; N-NCR, N-terminal signal peptide of NCRs; UPS, Ureide Permease; VPE, vacuolar processing enzyme.

The novelty of nodule development is the ability for cells within the nodule to accommodate intracellular bacteria and the activation of processes that create a suitable environment for nitrogen fixation. Understanding these processes is central to the engineering of the nitrogen fixation state. Multiple legumes and all actinorhizal nodulators have “fixation threads” that contain either filamentous *Frankia* bacteria or single-celled rhizobia, within a continuous infection thread that proliferates within the cells of the nodule, showing strong parallels to an arbuscule in the mycorrhizal symbiosis. Many species of legumes have taken this process a step further, with release of rhizobia into membrane-bound compartments contained within the cells of the nodule, so-called symbiosomes. Recent work suggests this innovation may have allowed an evolutionarily stabilising effect for nitrogen fixation in legumes, perhaps by providing much greater host-control of the bacterial symbionts [96]. A further step in host-control of symbionts observed in some legumes is the terminal differentiation of rhizobia into bacteroids, a state that cannot be reversed to free-living bacteria [96,126]. While these latter processes probably aid in the avoidance of cheaters and increase the efficiency of nitrogen fixation and its delivery, they are not essential, since plant species exist that fix nitrogen without either symbiosomes or bacteroids (Fig 4). While these latter stages may have desirable benefits, when considering the engineering of nitrogen fixation, they appear unessential. Thus, at least at this stage in the engineering process, they should not be the focus. What is critical for engineering nitrogen fixation is an understanding of the cellular state that allows intracellular colonisation by bacteria and the processes that facilitate the maturation of a nodule into a nitrogen-fixing structure, able to efficiently deliver fixed nitrogen to the host plant.

Alongside the activation of nodulation, the master regulator *NIN* with its close homologue *NLP2* controls the latter stages of nodule maturation, transitioning the nodule into the nitrogen-fixing state [133,134]. This late stage of NIN functionality involves the cleavage of NIN, liberating the C-terminal domain of NIN to directly activate late-stage genes associated with nitrogen fixation [133]. Alongside the induction of peptides that drive bacteroid differentiation [135] is the induction of leghemoglobins, which buffer oxygen to create a hypoxic environment for nitrogenase [134]. In parallel to these processes, tight control of metabolites needs to be engendered on the membrane that surrounds the bacteria, allowing exchange of a carbon supply, alongside elements essential for nitrogen fixation, in exchange for ammonia derived from nitrogenase action [136–138].

### Unsolved mystery 6: How do plant cells accommodate bacteria intracellularly?

Intracellular accommodation of bacteria provides a stable environment for nitrogen fixation. To accommodate the rhizobia, defence responses need to be suppressed, and the regulation of the cell cycle appears critical [104,139]. NIN suppresses the defence responses by regulating several genes that appear to function in the regulation of plant defences in the nodule: *Defective in Nitrogen Fixation 2* (*DNF2*), *Nodules with Activated Defence 1* (*NAD1*), and *Symbiotic Cysteine-rich Receptor Kinase* (*SymCRK*) [140–142]. The genetic characterisation of these genes implies a pathway functioning in the nodule associated with the regulation of plant defences, but the detailed mechanisms of how this pathway functions are unknown. Controlling endoreduplication in nodule meristematic cells has been shown to be essential for accommodating and maintaining rhizobia, with CCS*52A* playing a vital role in this control of the cell cycle and the production of polyploid cells [143]. It remains to be shown whether the polyploid nature of nodule cells and the down-regulation of plant defences alone are sufficient to allow intracellular bacterial accommodation. We need to know whether additional processes are essential for this accommodating state of nodule cells. Further, we need to understand the exact regulators that function in this late-stage maturation of a nodule, if that is more than *NIN* alone, to coordinate these processes, if we are to transfer this stable accommodation of nitrogen-fixing bacteria.

Transporting fixed nitrogen from the bacteria to the peribacteroid space may occur via diffusion or through protein channels [137]. An H^+^-ATPase on the symbiosome membrane pumps H^+^ into the peribacteroid space creating an acidic environment, which traps ammonium by protonating ammonia, producing ammonium cations. The down-regulation of a rhizobial ammonium transporter, AMT, might be a mechanism to prevent ammonium from flowing back into the bacteroid [144]. Ammonia and ammonium cations are then exported to the plant cytoplasm via two transporters on the symbiosome membrane: a voltage-activated monovalent cation channel [145] and nodulin 26 (NOD26) [146]. The cation channel is nonselective and can transport NH_4_^+^, K^+^, and Na^+^. Whether NH_4_^+^ specific channels are also located on this membrane is currently unknown. NOD26 is an aquaglyceroporin and can transport H_2_O and NH_3_ [146] and associates with glutamine synthetase (GS), which rapidly assimilates NH_3_ into glutamine (Gln), creating a sink for NH_3_ transport. Exported ammonia or ammonium cations can also be assimilated into glutamate (Glu) by glutamate synthase, transferring the amide group from Gln to α-ketoglutarate. Depending on the legume species and nodule types, Gln and Glu are converted into Asparagine (Asn) or Ureides for long-distance transport through the xylem [138]. Ureide Permeases in *Phaseolus vulgaris* [147] and in *Glycine max* [148] have been demonstrated on membranes of nodule cortical cells and vascular cells, and ship ureides into the xylem (Fig 4).

The last stage of nodule organogenesis is senescence, which plays a vital role in regulating the nodule nitrogen fixation function in response to ageing or environmental signals [149–151]. Nodule senescence leads to the disintegration of bacteroids and host plant cells and ends this symbiotic association. One of the most critical aspects of this senescence process is a rise in proteolytic activity. Previous studies have shown that different cysteine proteinases (CPs) are highly expressed in nodules at the senescence stage, especially a papain peptidase (CP6) and a vacuolar processing enzyme (VPE) [152]. Both their expressions and activities are increased during nodule senescence induced by abiotic stress or age [150–152]. The early expression of *CP6* and *VPE* promotes senescence and serves as a marker for nodule senescence (Fig 4).

### Unsolved mystery 7: What controls nodule senescence?

Though progress has been made, detailed mechanisms and critical regulators involved in nodule senescence are not fully understood. One crucial aspect of nodule senescence is regulating CPs, which are controlled by a transcription factor NAM/ATAF/CUC (NAC)-encoding 969 (NAC969) [153] (Fig 4). Understanding the detailed mechanism of nodule senescence is important, since it allows the control of this association, ensuring it delivers benefits to the plant.

## Conclusions and future perspectives

Our understanding of the nitrogen-fixing symbiosis of legumes has advanced dramatically over the last few decades. This has provided us with detailed frameworks for how rhizobial bacteria are recognised, how bacterial infection is initiated, the developmental programs underpinning nodulation, and the processes that allow these structures to support nitrogen fixation and deliver its products to the plant. There appear to be very few novelties in the rhizobial symbiosis of legumes, with many genes underpinning this process being derived from the preexisting symbiosis with arbuscular mycorrhizal fungi and from root development. Rather than novel emergence of new genes, the evolution of nodulation appears to have involved the renetworking of preexisting processes, principally controlled by the master regulator *NIN*. This transcription factor appears at all stages of the development of the nitrogen-fixing nodule, and perhaps more than anything, we need to understand not only that this transcription factor can control all of these steps, but how it is able to activate such diverse development in different cell types. Key to this question is a high degree of cellular resolution of the developmental processes associated with nodulation. The emergence of spatial omics, such as single-cell sequencing, as well as spatial transcriptomics [154] will facilitate such resolution. The concept of renetworking preexisting processes creates a realistic challenge for transferring nitrogen fixation: consider this over building entire signal transduction pathways or developmental processes from scratch. Even so, this remains a significant challenge. However, if the field continues to advance at the pace it has in the last two decades, then we hope a solution that delivers secure, sustainable, and affordable food will be in reach within the next decade.

## Abbreviations

AKG: α-ketoglutarate
AMT: ammonium transporter
Asn: Asparagine
CaM: calmodulin
CCaMK: calcium and calmodulin-dependent protein kinase
CP: cysteine proteinase
*DMI*: *DOES NOT MAKE INFECTIONS*
*DNF*: *DEFECTIVE IN NITROGEN FIXATION*
ENOD11: EARLY NODULIN11
ERN1: Ethylene Response Factor Required for Nodulation1
FLOT4: FLOTILLIN 4
GEF: Guanine-nucleotide Exchange Factor
Gln: glutamine
Glu: glutamate
GRP: glycine-rich peptide
GS: glutamine synthetase
*IPD3*: *INTERACTING PROTEIN OF DMI3*
*LBD16*: *LATERAL ORGAN BOUNDARIES DOMAIN 16*
LCO: lipochitooligosaccharide
LIN: LUMPY INFECTION
Lj: *Lotus japonicus*
LRR: Leucine-Rich Repeats
LysM: lysine motif
Mt: *Medicago truncatula*
NAC: NAM/ATAF/CUC
NAC969: NAC-encoding 969
*NAD1*: *Nodules with Activated Defence 1*
NCR: nodule-specific cysteine-rich peptides
NF: Nod factor
*NF-Y*: *Nuclear Factor-Y*
NIN: NODULE INCEPTION; nod, nodulation
NOD26: nodulin 26
*NOOT*: *NODULE ROOT*
NPL: NODULE PECTATE LYASE
*RAM1*: *REDUCED ARBUSCULAR MYCORRHIZA1*
RBOH: Respiratory Burst Oxidase Homolog
RIP: receptor-interacting protein
RNS: root nodule symbiosis
RPG: RHIZOBIUM-DIRECTED POLAR GROWTH
SCAR/WAVE: Suppressor of cAMP receptor defect/WASP family verpolin homologous protein
*SHI/STY*: *SHORT-INTERNODES/STYLISH*
*SYFO1*: *SYMBIOTIC FORMIN 1*
*SymCRK*: *Symbiotic Cysteine-rich Receptor Kinase*
UPS: Ureide Permease
VPE: vacuolar processing enzyme
VPY: VAPYRIN
